# The impact of polytrauma on sRAGE levels: evidence and statistical analysis of temporal variations

**DOI:** 10.1186/s13017-019-0233-6

**Published:** 2019-03-18

**Authors:** Lukas L. Negrin, Robin Ristl, Gabriel Halat, Thomas Heinz, Stefan Hajdu

**Affiliations:** 1 Department of Orthopedics and Trauma Surgery, Medical University of Vienna, Waehringer Guertel 18-20, 1090 Vienna, Austria; 2 Center for Medical Statistics and Informatics, Medical University of Vienna, Waehringer Guertel 18-20, 1090 Vienna, Austria

**Keywords:** Polytrauma, Thoracic trauma, Soluble receptor of advanced glycation end products, sRAGE, Biomarker

## Abstract

**Background:**

According to recently published findings, levels of the soluble receptor of advanced glycation end products (sRAGE) and its clearance from the blood may reflect the evolution of lung damage during hospitalization. Thus, the objective of this study was to reveal the course of sRAGE levels over the first three posttraumatic weeks, focusing on the severity of thoracic trauma and the development of acute respiratory distress syndrome (ARDS) and/or pneumonia.

**Methods:**

Twenty-eight consecutive surviving polytraumatized patients suffering thoracic trauma, age ≥ 18 years, Injury Severity Score ≥ 16, and directly admitted to our level I trauma center were enrolled in this prospective study. Blood samples were taken initially and on days 1, 3, 5, 7, 10, 14, and 21 during hospitalization. Luminex multi-analyte-technology was used for biomarker analysis.

**Results:**

Common to all our patients was an almost continuous decline of sRAGE levels within the first five posttraumatic days. Day 0 levels in polytrauma victims with severe thoracic trauma were more than twice as high than in those suffering mild thoracic trauma (*p* = 0.035), whereas the difference between the two groups did not reach significance from day 1. Neither the development of ARDS and/or pneumonia nor the necessity of secondary surgery did result in significant differences in sRAGE levels between the subgroups with and without the particular complication at any time point.

**Conclusions:**

sRAGE levels assessed immediately after hospital admission might serve as a diagnostic marker for the vehemence of impacts against the chest and thus might be applied as an additional tool in diagnosis, risk evaluation, and choice of the appropriate treatment strategy of polytraumatized patients in routine clinical practice.

## Background

The receptor of advanced glycation end products (RAGE) is a multiligand cell-surface protein belonging to the immunoglobulin (Ig) superfamily [1]. RAGE is composed of a single hydrophobic transmembrane domain, a highly charged cytosolic intracellular tail, and a large extracellular ligand-binding region comprising three Ig-like domains [2]. This membrane-bound form of RAGE is termed full-length RAGE (flRAGE) [3]. In the circulation of humans, soluble RAGE (sRAGE) has been identified, having the same extracellular domain and thus the same ligand-binding specificity as flRAGE but lacking the transmembrane and cytoplasmic domains [4]. sRAGE is comprised of two distinct isoforms. Cleaved RAGE (cRAGE) is produced via proteolytic cleavage of flRAGE at the cell surface by matrix metalloproteases and endogenous secretory RAGE (esRAGE) results from alternative splicing from a truncated RAGE pre-mRNA [3, 5, 6]. In the majority of healthy adult tissues, RAGE is expressed at a low basal level, whereas pulmonary tissues express remarkably high basal levels of RAGE [7], particularly on alveolar type I epithelial [8] and endothelial cells [9].

Recently, we identified sRAGE levels, measured shortly after the trauma occurred, as a promising diagnostic tool for assessing the severity of parenchymal lung injury in polytraumatized patients [10]. Our findings led us hypothesize that sRAGE levels might also indicate the evolution of lung damage during hospitalization. Thus, the objective of this study was to reveal the time-dependent course of sRAGE levels over the first three posttraumatic weeks, focusing on the severity of thoracic trauma and the development of acute respiratory distress syndrome (ARDS) and/or pneumonia, which both have a great impact on the treatment regimen of polytraumatized patients.

## Methods

Twenty-eight consecutive polytraumatized patients (1) not less than 18 years of age, (2) with an Injury Severity Score (ISS) of at least 16, (3) who suffered blunt thoracic trauma (Abbreviated Injury Scale (AIS) thorax ≥ 1), (4) who were directly admitted to our level I trauma center, (5) who were transferred to the intensive care unit (ICU) after initial treatment, and (6) who survived their injury were enrolled in this prospective study, which was approved by the local ethics committee. Burn victims and patients with known history of malignancies, asthma (stages 3/4), pulmonary fibrosis, pneumonitis, COPD Gold III/IV, auto-immune disorders, previous organ transplantation, or ongoing immunosuppressive therapy were excluded. Ten healthy adults, who had responded our call for volunteers, were combined to our control group. Only one blood sample was taken from them.

During the initial assessment and diagnostics, together with the routine venous blood samples, one separating gel tube (Vacuette® 8 ml; Greiner Bio-One International) was withdrawn from each polytraumatized patient for biomarker level measurement. Immediately afterwards, this additional sample was centrifuged at 3000*g* for 15 min at room temperature. After this procedure, serum was removed and stored at − 80 °C until assayed. Blood samples for biomarker level measurement were taken again on day 1, day 3, day 5, day 7, day 10, day 14, and day 21 during hospitalization as long as the patient consented. For biomarker analyses, we used Luminex multi-analyte technology (R & D systems Magnetic Luminex® Screening Assay – human Premixed Multi-Analyze Kit Number LXSAHM). We performed all measurements in technical duplicates and calculated the respective mean values. Our patients were informed about blood sampling at the earliest time point possible. In case written consent was refused, no further blood samples were taken and the already acquired material was destroyed if requested by the patient.

ARDS was diagnosed according to the Berlin definition [11] that characterizes ARDS by timing, chest imaging, origin of edema, and oxygenation. Clinical evidence for the diagnosis of pneumonia included an abnormal temperature (> 38 °C or < 35.5 °C); either leukocytosis (white cell count > 10,000/mm^3^ or > 10% immature forms) or leucopenia (white cell count < 4000/mm^3^); a macroscopically purulent sputum; the presence of a new cough, dyspnea, and/or tachypnea (in the case of spontaneous breathing patients); and a new or changing infiltrate on chest radiograph.

Statistical analysis was performed using the statistical software R 3.5. [12]. Demographic data are presented as mean and range in square brackets. sRAGE levels in text and graphics are displayed as mean ± standard error of the mean (SEM). If two SEM error bars did not overlap in the diagram when comparing two groups, a *t* test was used to determine if the means were significantly different at this specific time point. To test the null hypothesis of overall equality of the time trajectories in the first week from admission, comprising days 0, 1, 3, 5, and 7 with completely observed data for all subjects, the minP permutation test by Westfall and Young [13] was used with 20,000 random permutations. The test takes the correlation of repeated measurements within each subject into account and is appropriate for small sample sizes. The within-subject correlation of sRAGE levels across time was assessed by means of Pearson’s correlation coefficient. The Mann-Whitney *U* test was applied to compare continuous variables, whereas categorical data were analyzed by means of the chi-square test. The Spearman’s rank correlation coefficient was used to test the association between day 0 levels of sRAGE and selected parameters. In general, a *p* value of < 0.05 was considered significant.

## Results

Twenty-one males and seven females (age, 38.4 [18–85] years; ISS, 35.1 [21–50]) formed our study group. Their demographic data are presented in Table 1. Of interest, a moderate negative correlation between the day 0 levels of sRAGE and the base excess at admission could be calculated (Spearman’s *ρ* = − 0.466; *p* = 0.012), whereas no significant associations between the day 0 levels of sRAGE and the parameters shock index (*ρ* = 0.298; *p* = 0.124), lactate (*ρ* = 0.329; *p* = 0.087), and hemoglobin (*ρ* = − 0.315; *p* = 0.102) at admission could be revealed. ARDS was diagnosed in ten of the 11 affected patients within the first three posttraumatic days, whereas it developed in patient nine on day 10. First signs of pneumonia were clearly distinct not earlier than on day 4 after admission. Secondary surgery was performed in nine polytrauma victims (Table 2). No significant differences in ISS, length of stay in the ICU, number of ventilation days, and the incidence of pneumonia could be observed between patients undergoing and not undergoing subsequent surgeries (*p* ≥ 0.332), whereas the length of inpatient stay until discharge home or to another health care facility (59.3 [30–97] versus 38.9 [9–97]) and the incidence of ARDS (6 versus 5) were higher in those patients, who had to experience repetitive surgical treatment (*p* = 0.028; *p* = 0.041).

**Table 1:** Demographic data

Patient number	Gender	Age	Cause of injury	Transfer	Shock index at admission	Day 0 levels of sRAGE (pg/mL)	Lactate (mmol/L) at admission	Base excess (mmol/L) at admission	Hemoglobin (g/dL) at admission	Intubation on site	Chest drain	ECMO	Hemofiltration	Ventilation days	Stay at the ICU (days)	Inpatient stay (days)	ISS	AIS_Head_≥ 3	AIS_Thorax_	Rib fractures	Flail chest	Sternum fracture	Thoracic spine fracture	Diaphragmatic rupture	Aortic dissection	Lung contusion	Lung laceration	Pneumo/hemothorax	Pneumomediastinum	Hypothermia (< 32 °C)	ARDS	Pneumonia
1	m	41	F	A	1.04	3706	2.3	− 2.6	14.5					41	44	97	29	y	2							u					y	y
2	m	18	P	A	0.7	4267	1.2	− 3.1	11.7	y				11	14	37	30	y	1													y
3	m	20	P	A	0.6	6360	2.5	− 2.9	13.4					10	16	19	26	y	2							u						
4	f	26	V	H	0.8	40, 447	3.0	− 5.7	10.2	y	y	y		11	21	21	43	y	5	≥ 3 u			y			b	u	b	y		y	
5	f	61	P	A	1.1	7227	3.0	− 7.7	9.4	y				6	13	28	34		5	≥ 3 b	y					b		u				y
6	f	39	P	H	0.6	2823	0.8	− 1.6	9.4	y				31	40	55	34	y	1													y
7	m	21	F	A	0.8	2004	4.1	− 7.4	8.2	y				14	21	97	34		2							u					y	
8	f	21	V	H	0.8	3066	3.8	− 13.4	5.8	y				15	31	92	50		5	≥ 3 b						u		u			y	
9	m	27	O	H	0.9	12,940	5.3	− 14.1	6.7				y	12	28	72	34		1												y	y
10	m	51	P	A	0.7	1577	2.6	− 4.1	13.2	y				9	12	23	21	y	2							u						y
11	f	32	P	A	0.78	16,399	1.2	− 2.1	10.9		y			16	19	50	34		3	≥ 3 u	y	y				u		u				
12	m	43	V	H	0.7	5220	2.1	− 4.0	13.1	y	y			8	16	30	33		5	≥ 3 b	y	y		y		b					y	y
13	m	48	V	H	0.9	4763	2.4	− 1.4	13.4	y				42	49	59	33	y	4							b					y	y
14	f	40	V	H	0.7	2743	2.1	− 4.8	10.3	y	y			2	8	24	50		5	≥ 3 b	y	y	y			b	u	b				
15	m	65	F	H	0.5	12,696	2.3	− 0.4	13.1					4	8	62	41	y	4				y			u						
16	m	18	P	A	0.9	7518	3.8	− 6.2	9.2					12	18	22	41	y	4	1						b	u	u			y	
17	m	20	P	A	0.5	10,642	4.8	− 6.1	10.6	y	y			24	33	60	45	y	4				y			u		u				y
18	m	79	V	A	1.0	6963	1.2	− 4.0	10.8	y				10	15	16	22		2			y				u						
19	m	49	V	A	0.8	3086	4.0	− 2.9	13.0	y				1	10	21	22		3	≥ 3 u						u						
20	m	22	V	H	1.0	3066	3.9	− 4.6	7.2	y				6	9	9	48	y	4	2 u						b						
21	m	23	V	H	1.3	1239	4.1	− 8.0	11.6	y	y			30	43	85	45		5	≥ 3 u					y	b		u			y	y
22	m	25	V	A	0.8	5020	2.5	− 3.4	11.5					1	9	46	34		3	1		y				b						
23	m	75	F	H	0.7	2787	2.6	− 3.2	13.4		y			7	24	38	27	y	3	≥ 3 u						b	u	u				
24	f	35	P	A	0.6	3451	1.7	− 5.7	10.6	y				9	17	45	34	y	2	1						u						
25	m	41	V	H	0.9	6872	7.5	− 18.0	11.8	y	y			16	25	62	34		4	1		y				b		u			y	y
26	m	32	F	A	1.3	4848	1.6	− 4.0	13.3		y			9	18	18	41		3	≥ 3 u						u		u				
27	m	85	F	H	0.48	2553	0.8	− 2.5	15.7					18	42	43	30	y	1													y
28	m	18	R	A	1.0	12,625	13.7	− 19.1	10.2	y	y	y		9	21	42	35		5							b		b	y	y	y	

“No” and “nonexistent” are indicated by blank fields

*m* male, *f* female, *F* fall from a height ≥ 2 m, *P* vehicle-, streetcar-, or train-pedestrian collision, *V* vehicle accident, *O* overrun by wheel loader, *R* raid, *A* ambulance, *H* emergency rescue helicopter, *u* unilateral, *b* bilateral, *y* yes

**Table 2:** Subsequent surgeries

Patient number	Day													
	1	2	3	4	5	7	9	10	11	12	14	15	20	21
7			Soft tissue revision				External fixator pelvis and foot					Soft tissue revision		Humeral fracture plating Spine stabilization
8		Soft tissue revision				Soft tissue revision				Soft tissue revision				
9	Soft tissue revision		Soft tissue revision		Soft tissue revision	Soft tissue revision		Soft tissue revision					Soft tissue revision	
11				Laparotomy, splenectomy										
12								Bilateral humeral fracture plating						
22									Symphyseal plating					
25					Soft tissue revision				Soft tissue revision					
27											Tracheostomy			
28								Hematoma evacuation orbital cavity						

**Fig. 1:**
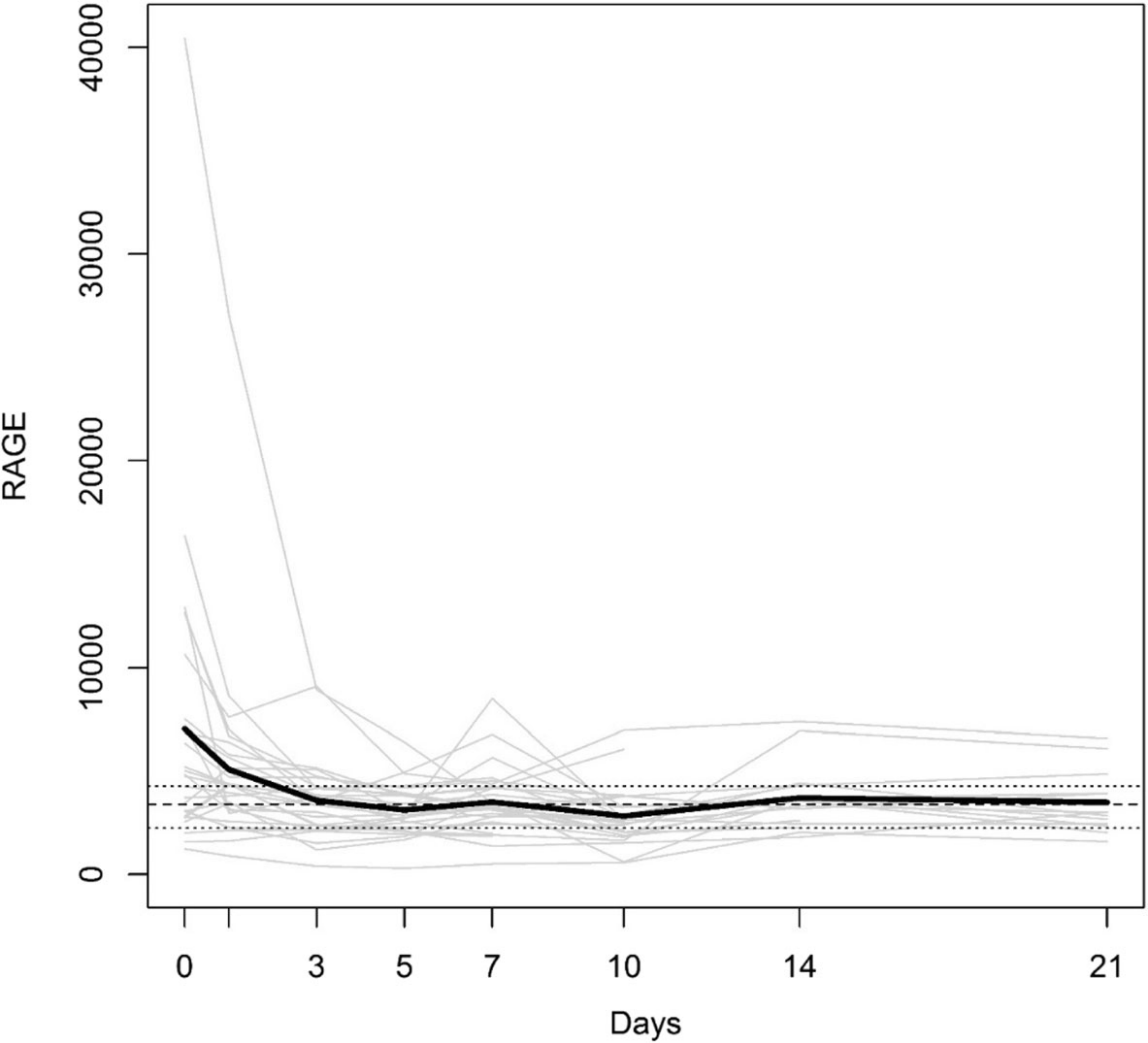
Individual and mean time-dependent courses of sRAGE. Thin gray lines represent the individual courses, whereas the mean course is shown as black bold line. The dotted line refers to the mean sRAGE level of the healthy control group, and its range is displayed by the two dashed lines

To investigate the natural history of sRAGE levels during the first 3 weeks following polytrauma, 28 samples were available up to and including day 7, whereas only 25, 17, and 13 samples, respectively, could be analyzed on days 10, 14, and 21, determined by the date of discharge from hospital and/or the patient’s willingness. Figure 1 shows the mean time course of sRAGE levels (black bold line) within the first 21 posttraumatic days. The mean sRAGE level of the healthy control group (3404 ± 237 pg/mL) is used as a reference value, indicated by the dotted line, whereas its range (2245–4292 pg/mL) is displayed by the two dashed lines. In the study group, the mean day 0 level amounts to 7032 ± 1443 pg/mL, being twice as high than the reference value, falling into its range within 3 days, where it remains for the entire study period. Additionally, Fig. 1 provides the sRAGE levels of each individual over time. Their time courses seem to be largely consistent from day 0 to day 5. This assumption is confirmed by the fact that any two sRAGE levels are highly correlated within this period (Table 3).

**Table 3:** Correlation coefficients between sRAGE levels

	Day 0	Day 1	Day 3	Day 5
Day 0	1	0.942**	0.697**	0.738**
Day 1	0.942**	1	0.742**	0.766**
Day 3	0.697**	0.742**	1	0.861**
Day 5	0.738**	0.766**	0.861**	1

**A significant correlation at a level of 0.01 (two-sided)

According to our objective, we subdivided our patients into two groups, “Thorax 0”, combining 10 patients with mild thoracic trauma (AIS_Thorax_ < 3), and “Thorax 1”, comprising 18 patients with severe thoracic trauma (AIS_Thorax_ ≥ 3). “Thorax 1” versus “Thorax 0” did not differ in length of stay in the ICU (20.8 [8–49] days versus 24.9 [12–44] days; *p* = 0.494), in inpatient stay (42.7 [9–92] days versus 50.4 [16–97] days; *p* = 0.654), in ventilation time (12.2 [1–42] days versus 16.3 [9–41] days; *p* = 0.146) and in the incidence of ARDS (8 versus 3, *p* = 0.453) and pneumonia (6 versus 6; *p* = 0.172). Not surprisingly, the number of lung contusions (18 versus 6; *p* = 0.001) and the ISS (38.3 [22–50] versus 29.4 [21–34]; *p* = 0.004) were higher in the “Thorax 1” group.

**Fig. 2:**
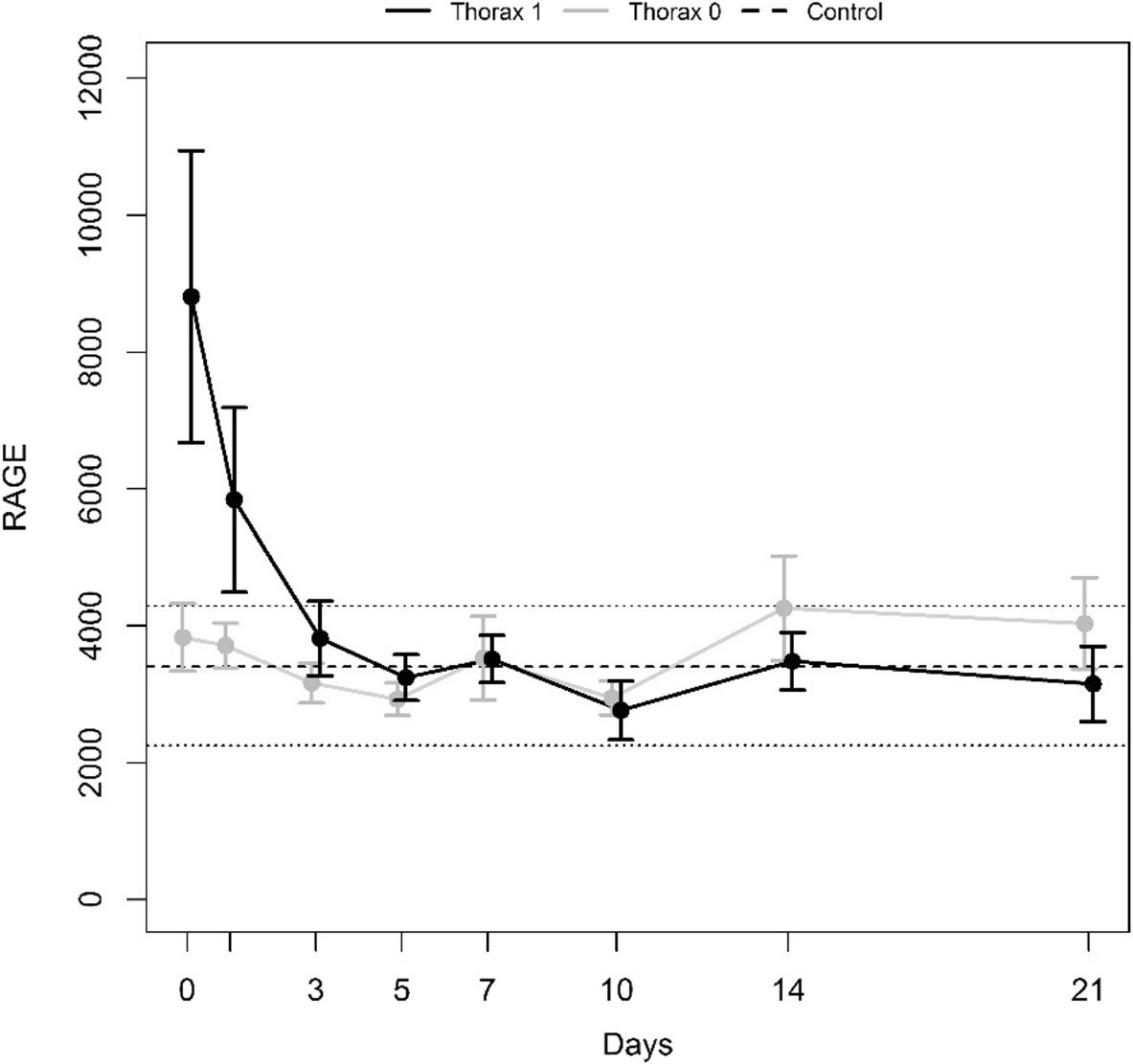
Mean time courses with standard error of the mean of the subgroups “Thorax 1” and “Thorax 0.” The dotted line refers to the mean sRAGE level of the healthy control group, and its range is displayed by the two dashed lines.

The time courses of “Thorax 1” and “Thorax 0” are presented in Fig. 2. At admission, the mean sRAGE level was significantly higher in “Thorax 1” compared to “Thorax 0” (8812 ± 2134 pg/mL versus 3829 ± 1559 pg/mL; *p* = 0.035), whereas no significant difference was revealed for the day 1 levels (5842 ± 1348 pg/mL versus 3712 ± 329 pg/mL; *p* = 0.1412). The minP test provided *p* = 0.0759 for the first week from admission.

**Fig. 3:**
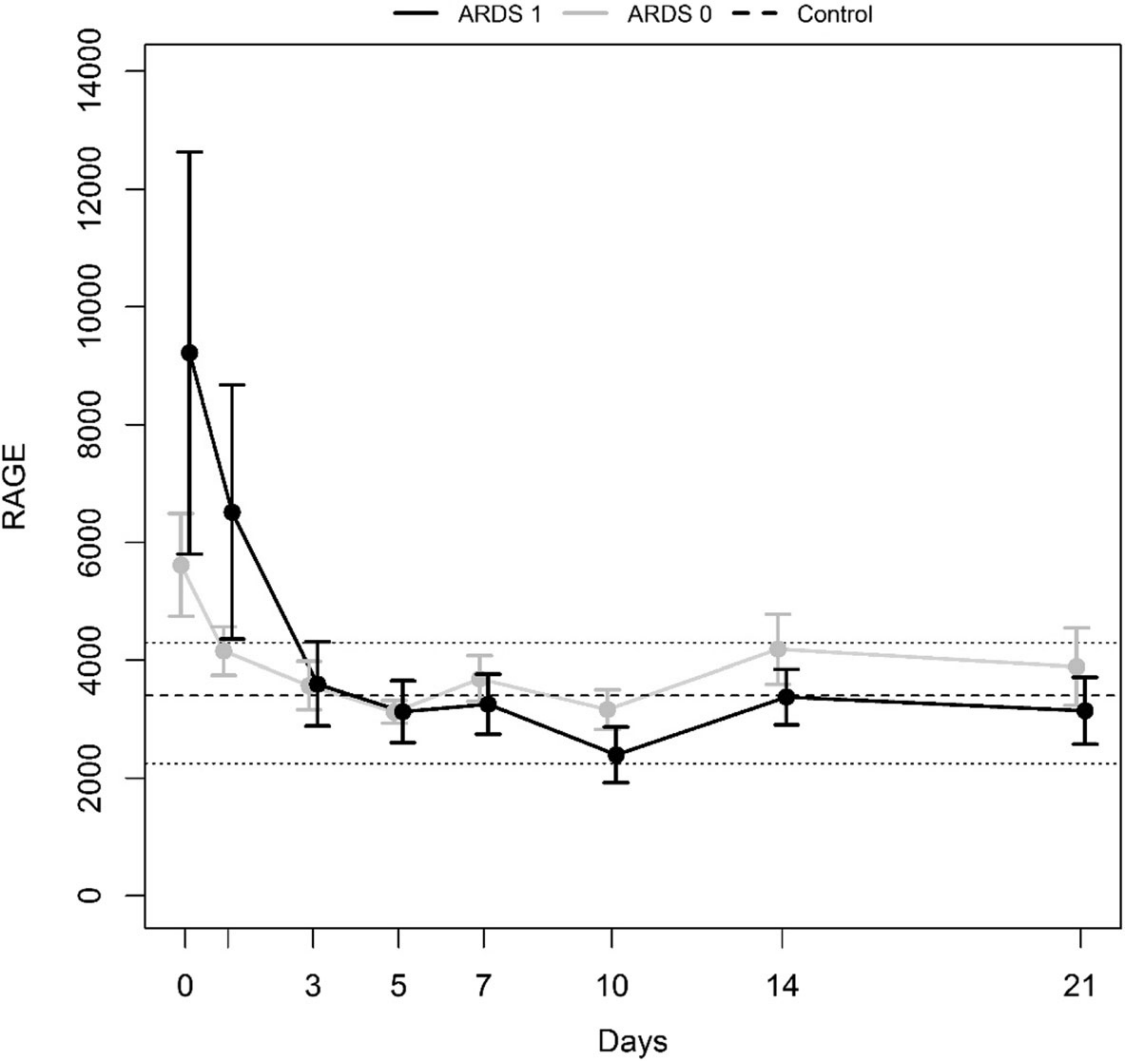
Mean time courses with standard error of the subgroups “ARDS 1” and “ARDS 0”. The dotted line refers to the mean sRAGE level of the healthy control group, its range is displayed by the two dashed lines

“ARDS 1” combined all patients developing ARDS, whereas “ARDS 0” referred to those without ARDS. The relevant time courses are shown in Fig. 3; *p* = 0.7053 was computed for the minP test.

Furthermore, group comparisons by means of the minP test were calculated with regard to the occurrence of pneumonia (*p* = 0.1588) and the necessity of secondary surgery (*p* = 0.2697). Of interest, no distinct changes in sRAGE levels after subsequent surgery could be observed.

## Discussion

Our prospective study revealed an almost continuous decline of individual sRAGE levels within the first five posttraumatic days in all of our patients. Day 0 levels in polytrauma victims with severe thoracic trauma were more than twice as high than in those suffering mild thoracic trauma. However, the very next day, a significant difference in sRAGE levels between the two groups could not be detected anymore. Neither the development of ARDS and/or pneumonia nor the necessity of secondary surgery did result in significant differences in sRAGE levels between the subgroups, combining patients with and without the particular complication, at any time point.

All of our patients sustained blunt thoracic trauma, including lung contusions in 24 and concomitant lung lacerations in four patients. Lung contusions and lacerations are caused by a strong direct force to the thoracic cage [14], which is immediately transmitted to the lung parenchyma, resulting in the disruption of the epithelial and endothelial cell lining of the lung, alveolar hemorrhage, and interstitial extravasations of red cells and plasma [15, 16]. The damaged tissue activates systemic innate immune mechanisms with high local concentrations of proinflammatory mediators that stimulate chemotaxis and recruit neutrophils to the injured lung [17] resulting in an increase in cell membrane permeability and protein-rich alveolar edema [16] that might lead to the development of ARDS [18], more specifically to direct ARDS, as it is caused by direct injury to the lung epithelium [19]. Contrarily, in indirect ARDS, the vascular endothelium of the lung is diffusely damaged by circulating inflammatory mediators released in the setting of systemic disorders [19]. ARDS evolved in 11 patients of our study population. Whereas indirect ARDS may be assumed in patient 9 due to his injury pattern and his medical history, each of the ten remaining patients suffered a lung contusion, a trigger for direct ARDS.

The strength of the direct mechanical impact to the chest determines the severity of the thoracic injury, which is reflected in the AIS_Thorax_ value. Initial sRAGE levels are higher in patients suffering severe thoracic trauma than in patients with mild thoracic trauma, they respond rapidly after lung injury, and they indicate a clear temporal course within the first five posttraumatic days, with a significant decline from day 0 to day 1. Then, individual variations within the range of the reference value are observed. In our opinion, these findings suggest that it is the mechanical deterioration of the epithelium and endothelium that triggers the release of an abundant amount of sRAGE for a short period. sRAGE seems to be promptly cleared by the kidneys, as indicated by the rapid decline of its levels, especially in polytraumatized patients suffering severe thoracic trauma. Alternatively, stimuli generated during the subsequent pulmonary inflammatory response might trigger the sRAGE release. In this scenario, the development of ARDS should greatly affect sRAGE levels, as a profound role in the pathophysiology of ARDS is played by the innate immune response [20]. Although ARDS developed no later than the third posttraumatic day in 91% of the affected patients, its presence had neither an effect on value nor course of sRAGE levels within the first 5 days after the polytrauma occurred. Noteworthy, our findings are in line with the results of a recently published prospective study focusing on 103 polytraumatized patients with severe thoracic trauma, which did not reveal significant differences in sRAGE levels in individuals developing/not developing ARDS as well as in individuals suffering/not suffering pneumonia either [21].

## Conclusions

sRAGE levels assessed immediately after hospital admission seem to be a potential marker for the vehemence of impacts against the chest. If implemented in routine clinical practice, they might serve as an additional tool in diagnosis and risk evaluation and thus might help the trauma team in their decision-making on the appropriate treatment strategy in polytrauma victims. Hopefully, further research in large multicenter studies will verify our results and provide reliable cutoff values for quantifying the level of thoracic destruction.

## Abbreviations

AIS: Abbreviated Injury Scale
ARDS: Acute respiratory distress syndrome
cRAGE: Cleaved RAGE
esRAGE: Endogenous secretory RAGE
flRAGE: Full-length RAGE
ICU: Intensive care unit
ISS: Injury Severity Score
RAGE: Receptor of advanced glycation end products
sRAGE: Soluble RAGE
